# *dact1/2* modifies noncanonical Wnt signaling and *calpain 8* expression to regulate convergent extension and craniofacial development

**DOI:** 10.1101/2023.11.07.566024

**Published:** 2023-11-07

**Authors:** Shannon H. Carroll, Sogand Schafer, Kenta Kawasaki, Casey Tsimbal, Amélie M. Julé, Shawn A. Hallett, Edward Li, Eric C. Liao

**Affiliations:** 1 Center for Craniofacial Innovation, Children’s Hospital of Philadelphia Research Institute, Children’s Hospital of Philadelphia, PA 19104, USA.; 2 Division of Plastic and Reconstructive Surgery, Department of Surgery, Children’s Hospital of Philadelphia, PA 19104, USA.; 3 Shriners Hospital for Children, Tampa, FL 33607, USA; 4 Department of Biostatistics, Harvard T.H. Chan School of Public Health, Boston, MA 02115, USA

## Abstract

Wnt signaling plays a crucial role in the early embryonic patterning and development, to regulate convergent extension during gastrulation and the establishment of the dorsal axis. Further, Wnt signaling is a crucial regulator of craniofacial morphogenesis. The adapter proteins Dact1 and Dact2 modulate the Wnt signaling pathway through binding to Disheveled, however, the distinct relative functions of Dact1 and Dact2 during embryogenesis remain unclear. We found that *dact1* and *dact2* genes have dynamic spatiotemporal expression domains that are reciprocal to one another and to *wnt11f2l*, that suggest distinct functions during zebrafish embryogenesis. We found that both *dact1* and *dact2* contribute to axis extension, with compound mutants exhibiting a similar convergent extension defect and craniofacial phenotype to the *wnt11f2* mutant. Utilizing single-cell RNAseq and *gpc4* mutant that disrupts noncanonical Wnt signaling, we identified *dact1/2* specific roles during early development. Comparative whole transcriptome analysis between wildtype, *gpc4* and *dact1/2* mutants revealed a novel role for *dact1/2* in regulating the mRNA expression of the classical calpain *capn8*. Over-expression of *capn8* phenocopies *dact1/2* craniofacial dysmorphology. These results identify a previously unappreciated role of *capn8* and calcium-dependent proteolysis during embryogenesis. Taken together, our findings highlight the distinct and overlapping roles of *dact1* and *dact2* in embryonic craniofacial development, providing new insights into the multifaceted regulation of Wnt signaling.

## Introduction

Wnt signaling is a crucial regulator of embryogenesis through its regulation of body axis patterning, cell fate determination, cell migration, and cell proliferation (Logan and Nusse 2004, Steinhart and Angers 2018, Mehta, Hingole et al. 2021). Current mechanistic understanding of Wnt signaling during embryogenesis includes an extensive catalog of ligands, receptors, co-receptors, adaptors, and effector molecules (Clevers and Nusse 2012, Niehrs 2012, Loh, van Amerongen et al. 2016, Mehta, Hingole et al. 2021). The intricate spatiotemporal integration of Wnt signaling combinations is an important focus of developmental biology and tissue morphogenesis (Petersen and Reddien 2009, Clevers and Nusse 2012, Loh, van Amerongen et al. 2016, Wiese, Nusse et al. 2018). Disruptions of Wnt signaling-associated genes lead to a number of congenital malformations which often affect multiple organ systems given their pleotropic developmental functions (Hashimoto, Morita et al. 2014, Shi 2022). Craniofacial anomalies are among the most common structural congenital malformations and genes in the Wnt signaling pathway are frequently implicated (Ji, Hao et al. 2019, Reynolds, Kumari et al. 2019, Huybrechts, Mortier et al. 2020). The roles of certain Wnt signaling components may change in different temporal and spatial contexts, requiring detailed developmental and genetic analyses.

Genetic approaches in zebrafish have identified a number of key Wnt regulators of early development, with gastrulation and craniofacial phenotypes (Brand, Heisenberg et al. 1996, Hammerschmidt, Pelegri et al. 1996, Heisenberg, Brand et al. 1996, Piotrowski, Schilling et al. 1996, Solnica-Krezel, Stemple et al. 1996, Schilling and Le Pabic 2009). The *silberblick (slb)* mutant, later identified as a *wnt11f2* allele, exhibited gastrulation and midline craniofacial phenotypes that encompassed aspects of multiple mutant classes (Heisenberg, Brand et al. 1996). During early segmentation in the somite stage, the *wnt11f2* mutant developed a shortened anterior-posterior axis and partially fused eyes (Heisenberg, Brand et al. 1996). Subsequently as the cranial prominences converge and the anterior neurocranium (ANC) formed, instead of a fan-shaped structure observed in wildtype embryos, the *wnt11f2* mutant ANC appeared rod-like, with a significant deficiency of the transverse dimension (Heisenberg, Brand et al. 1996, Heisenberg and Nusslein-Volhard 1997). Another mutant *knypek*, later identified as *gpc4*, an extracellular Wnt co-receptor, was identified as a gastrulation mutant also with a shortened body axis (Solnica-Krezel, Stemple et al. 1996). In contrast, the *gpc4* mutant formed an ANC that is wider in the transverse dimension than the wildtype, in the opposite end of the ANC phenotypic spectrum compared to *wnt11f2* (Topczewski, Sepich et al. 2001, Rochard, Monica et al. 2016). These observations beg the question how defects in early patterning and convergent extension of the embryo may be associated with later craniofacial morphogenesis. The observation that *wnt11f2* and *gpc4* mutant share similar gastrulation and axis extension phenotypes but contrasting ANC morphologies supports a hypothesis that convergent extension mechanisms regulated by these Wnt pathway genes are specific to the temporal and spatial context during embryogenesis. As such, there may be Wnt signaling modifiers that act in specific temporal windows during development.

Dact (aka frodo, dapper) are scaffolding proteins that were identified to regulate Dishevelled (Dvl)-mediated Wnt signaling, both positively and negatively (Gloy, Hikasa et al. 2002, Waxman, Hocking et al. 2004, Gao, Wen et al. 2008, Wen, Chiang et al. 2010, Ma, Liu et al. 2015, Lee, Cheong et al. 2018). Dact proteins bind directly to Dvl (Gloy, Hikasa et al. 2002, Brott and Sokol 2005, Lee, Cheong et al. 2018) and have been found to interact with and inhibit members of TGFβ and Nodal signaling pathways (Zhang, Zhou et al. 2004, Su, Zhang et al. 2007, Meng, Cheng et al. 2008, Kivimae, Yang et al. 2011). Previous experiments using morpholinos to disrupt *dact1* and *dact2* in zebrafish found *dact1* morphants to be slightly smaller and developed a normal body axis (Waxman, Hocking et al. 2004). In contrast, *dact2* morphants were found to phenocopy described zebrafish gastrulation mutants, with impaired convergent extension, shortened body axis, and medially displaced eyes (Waxman, Hocking et al. 2004). By disrupting *dact1* and *dact2* and various wnt genes concurrently, it was concluded that *dact1* enhances wnt/β-catenin signaling, while *dact2* interacts with the wnt/PCP pathway (Waxman, Hocking et al. 2004).

Here we investigated the genetic requirement of *dact1* and *dact2* during embryogenesis and craniofacial development, using germline mutant alleles generated by CRISPR-targeted mutagenesis. We examined the connection between convergent extension governing gastrulation, body axis segmentation, and craniofacial morphogenesis. Differential single-cell RNA sequencing between wildtype, *dact1/2,* and *gpc4* mutants revealed distinct transcriptome profiles and the discovery of *calpain 8* (*capn8)* calcium-dependent protease to be ectopically expressed in the *dact1/2* mutants and functions to mediate Wnt signaling and craniofacial morphogenesis.

## Results

### *dact1* and *dact2* have distinct expression patterns throughout embryogenesis

**Figure 1.**
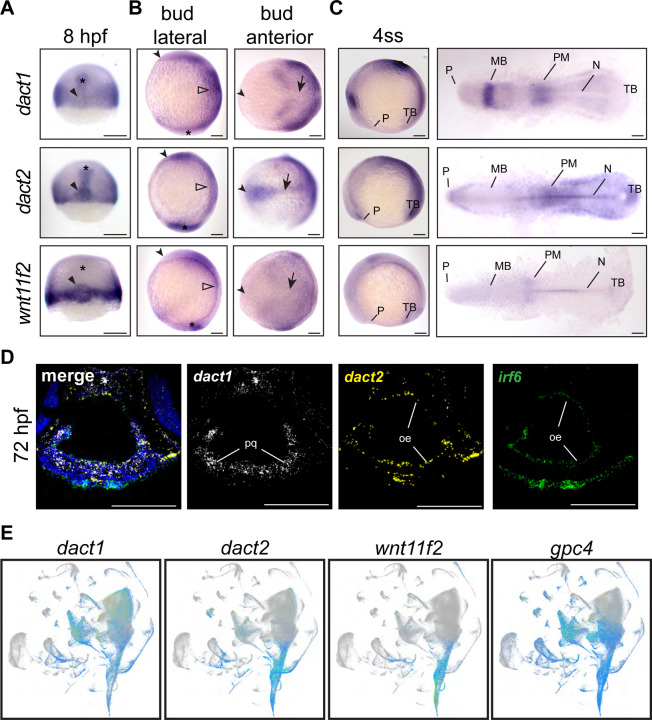
Unique and shared *dact1* and *dact2* gene expression domains during zebrafish development. **(A-C)** Whole-mount *in situ* hybridization showing *dact1*, *dact2,* and *wnt11f2* gene expression patterns. Scale bar = 100 μm. **A)** At 8hpf, *dact2* and *wntllf2* are highly expressed in the dorsal margin and presumptive Nieuwkoop center of the gastrulating embryo, with *dact1* being weakly detected (arrowhead). In contrast to *wnt11f2*, *dact1* and *dact2* are expressed in the presumptive dorsal mesoderm (asterisk). **B)** Lateral (anterior to the left of page) and anterior (dorsal side toward top of page) views of bud-stage embryos. *dact2* and *wnt11f2* transcripts are detected in the anterior neural plate (arrowhead) and tailbud (asterisk) while *dact2* is additionally expressed in the axial mesoderm (arrow). *dact1* gene expression is concentrated to the paraxial mesoderm (open arrowhead). **C)** Lateral and flat-mount views of 4 ss embryos. *dact2* is expressed in the anterior neural plate and polster (P), notochord (N), paraxial and presomitic mesoderm (PM) and tailbud (TB). *wnt11f2* is also expressed in these cells (Thisse 2001). In contrast, *dact1* is expressed in the midbrain (MP) and the paraxial and presomitic mesoderm. **D)** RNAscope *in situ* hybridization analysis of *dact1* (white) and *dact2* (yellow) and *irf6* (green) expression in transverse section of 72 hpf embryos. *dact1* is expressed in the orofacial cartilage (pq), while *dact2* is expressed in the oral epithelium (oe). The epithelial marker *irf6* is expressed in the oe and surface epithelium (se). Dapi (blue). Scale bar = 100 μm. **E)** Daniocell single-cell RNAseq analysis with a display of *dact1*, *dact2*, *wnt11f2* and *gpc4* clusters from 3–120 hpf of development (Farrell, Wang et al. 2018).

To determine the spatiotemporal gene expression of *dact1* and *dact2* during embryogenesis we performed wholemount RNA *in situ* hybridization (ISH) across key time points (Fig. 1) (Farrell, Wang et al. 2018, Lange, Granados et al. 2023). Given that the described craniofacial phenotypes of the *dact2* morphant and the *wnt11f2* mutant are similar (Heisenberg and Nusslein-Volhard 1997, Waxman, Hocking et al. 2004), we also performed *wnt11f2* ISH to compare to *dact1* and *dact2* expression patterns. Until spatial transcriptomics is widely applied for zebrafish, the wholemount ISH provides anatomic registry not available through scRNAseq atlas, though the latter is increasingly helpful to provide details on cell types expressing the genes of interest.

During gastrulation at 8 hours post-fertilization (hpf; 75% epiboly), some regions of *dact1* and *dact2* gene expression were shared and some areas are distinct to each *dact* gene (Fig. 1A). Further, *dact* gene expression was distinct from *wnt11f2.* Transcripts of *dact1*, *dact2* and *wntllf2* were all detected in the blastoderm margin, as previously described (Gillhouse, Wagner Nyholm et al. 2004). Transcripts of *dact2*, and to a lesser extent *dact1*, were also detected in the prechordal plate and chordamesoderm (Fig. 1A). Additionally, *dact2* gene expression was concentrated in the shield and presumptive organizer or Nieuwkoop center along with *wnt11f2*. This finding is consistent with previously described expression patterns in zebrafish and supports a role for *dact1 and dact2* in mesoderm induction and *dact2* in embryo dorsalization (Thisse 2001, Gillhouse, Wagner Nyholm et al. 2004, Muyskens and Kimmel 2007, Oteiza, Koppen et al. 2010). At the end of gastrulation and during somitogenesis the differences in the domains of *dact1* and *dact2* gene expressions became more distinct (Fig. 1B,C). At tailbud stage, *dact1* transcripts were detected in the trunk and the posterior paraxial mesoderm, whereas *dact2* transcripts were detected in the anterior neural plate, notochord, and tailbud, similar to *wnt11f2* gene expression. However, *dact2* was unique in that its expression demarcated the anterior border of the neural plate. As *dact2* morphants exhibited a craniofacial defect with medially displaced eyes and midfacial hypoplasia (Waxman, Hocking et al. 2004), we examined *dact1* and *2* expression in the developing orofacial tissues. We found that at 72 hpf *dact2* and the epithelial gene *irf6* were co-expressed in the surface and oral epithelium that surround the cartilaginous structures (Fig. 1D). This is in line with our prior finding of decreased *dact2* expression in *irf6* null embryos (Carroll, Macias Trevino et al. 2020). In contrast to *dact2*, *dact1* was expressed in the developing cartilage of the ethmoid and palatoquadrate of the zebrafish larvae (Fig. 1D).

The differences in expression pattern between *dact1* and *dact2* using publicly available Daniocell single-cell sequencing data (Farrell, Wang et al. 2018) (Fig. 1E). During embryogenesis, *dact2* was more highly expressed in anterior structures including cephalic mesoderm and neural ectoderm while *dact1* was more highly expressed in mesenchyme and muscle (Fig. 1E). We also utilized this tool to compare *dact1* and *dact2* expression to *wnt11f2* and *gpc4* expression, known noncanonical Wnt pathway members with craniofacial phenotypes (Heisenberg, Brand et al. 1996, Solnica-Krezel, Stemple et al. 1996, Heisenberg and Nusslein-Volhard 1997). In general, we found *dact1* spatiotemporal gene expression to be more similar to *gpc4* while *dact2* gene expression was more similar to *wnt11f2*. These results of shared but also distinct domains of spatiotemporal gene expression of *dact1* and *dact2* suggest that these paralogs may have some overlapping developmental functions while other roles are paralog specific.

### *dact1* and *dact2* contribute to axis extension and *dact1/2* compound mutants exhibit a convergent extension defect

*dact1* and *dact2* are known to interact with *disheveled* and regulate non-canonical Wnt signaling (Gloy, Hikasa et al. 2002, Waxman, Hocking et al. 2004, Gao, Wen et al. 2008, Wen, Chiang et al. 2010, Ma, Liu et al. 2015, Lee, Cheong et al. 2018). Previous work investigated the effect of *dact1* and *dact2* disruption during zebrafish embryogenesis using morpholinos and reported morphant phenotypes in embryonic axis extension and eye fusion (Waxman, Hocking et al. 2004). We were interested in how disruption of convergent extension in early embryogenesis may be related to anterior neurocranium (ANC) malformation, as has been described for *slb* (*wnt11f2*) and *dsh* mutants (Piotrowski, Schilling et al. 1996, Kimmel, Miller et al. 2001, Xing, Cheng et al. 2018). Further, we aimed to examine the genetic interactions between *dact1*/*dact2* and key Wnt regulators such as *wnt11f2*, *gpc4* and *wls*. These experiments required germline alleles in order to ensure the reproducibility of data and warranted generation of *dact1* and *dact2* alleles via CRISPR/Cas9 mediated targeted mutagenesis (Fig. S1). We generated a *dact1* mutant allele (22 bp deletion, hereafter *dact1*−/−) and a *dact2* allele (7 bp deletion, hereafter *dact2*−/−), both resulting in a premature stop codon and presumed protein truncation (Fig. S1). The specificity of the gene disruption was demonstrated by phenotypic rescue with injection of *dact1* or *dact2* mRNA (Fig. S1).

**Figure 2.**
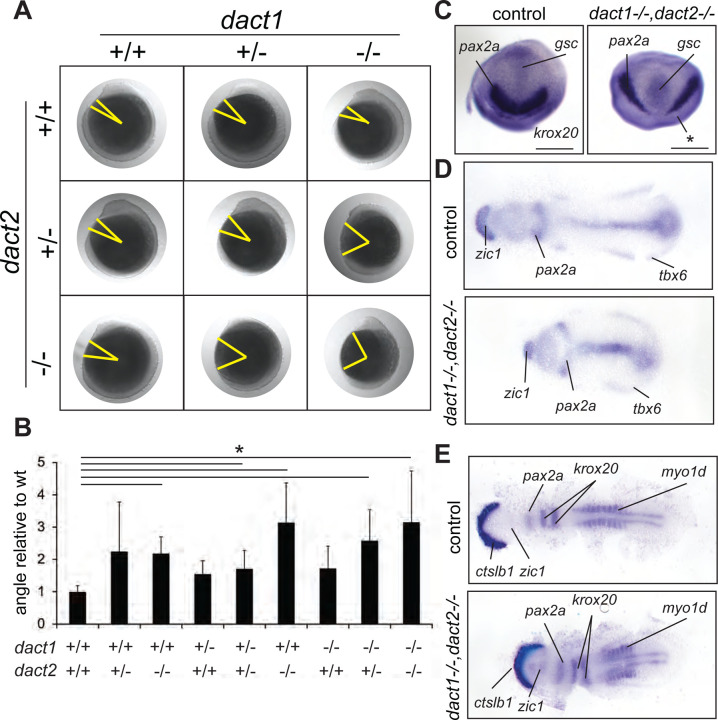
Impaired convergent extension in *dact1* and *dact2* compound mutants. **A)** Inter-cross of compound heterozygotes yield embryos with different degrees of axis extension that correspond to the *dact1* and *dact2* genotypes. Representative lateral images of embryos at 12 hpf. The yellow line indicates body axis angle measured from the anterior point of the head, the center of the yolk, to the end of the tail. **B)** Quantification of body axis angle. Numbers represent the difference in angle relative to the average wildtype embryo. Asterisk indicates genotypes with angles significantly different from wildtype. ANOVA p<0.5 n= 3–21 embryos. **C)** Representative bud stage wildtype and *dact/2*−/− mutant embryos stained for *gsc* (prechordal plate), *pax2a* (midbrain/hindbrain boundary) and *krox20* (rhombomere 3). Asterisk indicates lack of *krox20* expression in *dact1/2* mutant. Scale bar = 200 μm **D)** Representative flat mounts of 1–2 ss wildtype and *dact1/2* mutant embryos stained for *zic1* (telencephalon), *pax2a* and *tbx6* (ventrolateral mesoderm). **E)** Representative flat mounts of 10 ss wildtype and *dact1/2*−/− mutant embryos stained for *ctsl1b* (hatching gland), *zic1*, *pax2a*, *krox20*, and *myo1d* (somites).

Analysis of compound *dact1* and *dact2* heterozygote and homozygote alleles during late gastrulation and early segmentation time points identified embryonic axis extension anomalies (Fig. 2A). *dact1*−/− or *dact2*−/− homozygotes developed normally. However, at 12 hpf, *dact2*−/− single mutants have a significantly shorter body axis relative to wildtype. In contrast, body length shortening phenotype was not observed in *dact1*−/− homozygotes. Compound heterozygotes of *dact1*+/−; *dact2*+/− also developed normally but exhibited shorter body axis relative to wildtype. The most significant axis shortening occurred in *dact1*−/−; *dact2*−/− double homozygotes with a less severe truncation phenotype in the compound heterozygote *dact1+/−; dact2*−/− (Fig. 2A). Interestingly, these changes in body axis extension do not preclude the compound heterozygous larvae from reaching adulthood, except in the *dact1−/−; dact2*−/− double homozygotes which did not survive from larval to juvenile stages.

Body axis truncation has been attributed to impaired convergent extension (CE) during gastrulation (Tada and Heisenberg 2012). In order to delineate CE hallmarks in the *dact1*−/−;*dact2*−/− mutants, we performed whole-mount RNA ISH detecting genes that are expressed in key domains along the body axis (Fig. 2B). At bud stage, *dact1−/−; dact2*−/− embryos demonstrate bifurcated expression of *pax2a* and decreased anterior extension of *gsc* expression, suggesting impaired midline convergence and anterior extension of the mesoderm. At the 1–2 somite stage, *zic1*, *pax2a* and *tbx6* are expressed in neural plate, prospective midbrain and the tailbud, respectively, in both the wildtype and *dact1−/−; dact2*−/− embryos. However, the spacing of these genes clearly revealed the shortening of the anteroposterior body axis in the *dact1−/−; dact2*−/− embryos. Midline convergence is decreased and the anterior border of the neural plate (marked by *zic1* expression) was narrower in the *dact1−/−; dact2*−/− embryos (Fig. 2C). At the 10 somite stage (ss), *dact1−/−; dact2*−/− embryos showed decreased spacing between *ctslb1* and *pax2a* gene expression, suggesting impaired lengthening of the anterior portion of the embryo. Detection of muscle marker *myo1d* in the *dact1−/−; dact2*−/− embryos delineated impaired posterior lengthening as well as reduced somitogenesis, evidenced by the decreased number of somites (Fig. 2D). These data point to impaired CE of the mesoderm in *dact1−/−; dact2*−/− double mutants, which resulted in a shorter body axis. These findings were observed in a prior study using morpholinos to disrupt *dact1* and *dact2* expression, where CE defects were observed in *dact2* but not *dact1* or *dact1/2* double morphants (Waxman, Hocking et al. 2004). These *dact1*−/−;*dact2*−/− CE phenotypes were similar to findings in other Wnt mutants, such as *slb* and *kyn* (Heisenberg, Tada et al. 2000, Topczewski, Sepich et al. 2001).

### *dact1*/*dact2* compound mutants exhibit anterior neurocranium dysmorphology similar to *slb/wnt11f2* mutants

**Figure 3.**
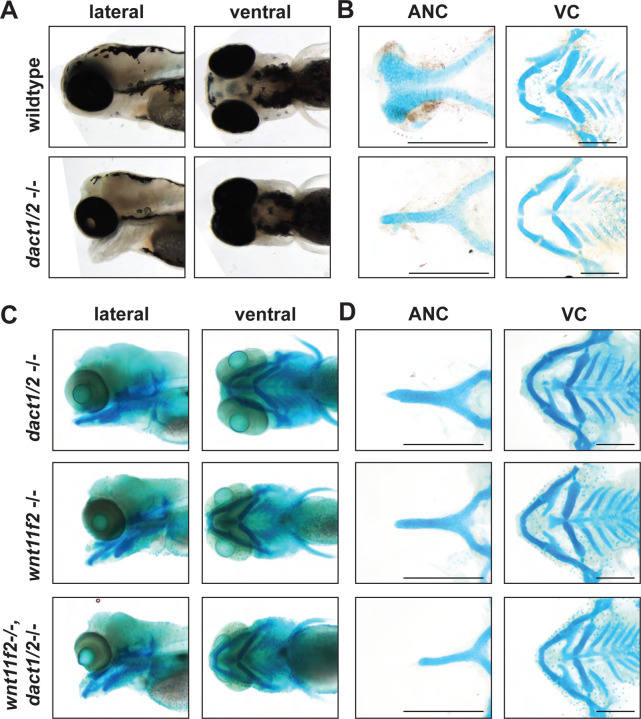
Midface development requires *dact1* and *dact2*. **A)** Representative brightfield images of wildtype and *dact1/2*−/− compound mutants at 4 dpf. Lateral and ventral views show d*act1/2*−/− compound mutants have a hypoplastic midface, medially displaced eyes, and a displaced lower jaw. **B)** Representative flat-mount images of Alcian blue stained ANC and VC elements from 4 dpf wildtype and d*act1/2*−/− compound mutants. D*act1/2*−/− mutants have a rod-shaped ANC with no distinct lateral and medial elements. No obvious differences were found in *dact1/2* mutant VC. **C)** Representative images of Alcian blue stained *dact1/2*−/−, *wntllf2−/−,* and *wnt11f2*−/−,*dact1*/*2*−/− compound mutants. Lateral and ventral views show similar craniofacial phenotypes in each mutant. **D)** Representative flat-mount images of Alcian blue stained ANC and VC elements show a similar phenotype between *dact1/2*−/−, *wntllf2−/−,* and *wnt11f2−/−,dact1/2*−/− compound mutants. Scale bar: 200 μm.

No craniofacial phenotype was observed in *dact1* or *dact2* single mutants (data not shown). However, in-crossing to generate *dact1−/−; dact2*−/− compound homozygotes resulted in dramatic craniofacial deformity (Fig. 3). Specificity of this phenotype to *dact1/2* was confirmed via rescue with *dact1* or *dact2* mRNA injection (Fig. S1). The *dact1−/−;dact2*−/− mutant embryos exhibited midfacial hypoplasia and the eye fields converged in the midline (Fig. 3A). The forebrain protruded dorsally and the mouth opening and ventral cartilage structures were displaced ventrally (Fig. 3A). Alcian blue staining of cartilage elements revealed severe narrowing of the anterior neurocranium (ANC) into a rod-like structure, while the ventral cartilage elements were largely unaffected (Fig. 3B). This *dact1−/−;dact2*−/− double mutant phenotype is highly similar to that described for *wnt11f2* (*slb*) mutants, a key regulator of non-canonical Wnt signaling and CE (Kimmel, Miller et al. 2001).

We hypothesized that the shared phenotypes between *wnt11f2* and *dact1/2* mutants point to these genes acting in the same signaling pathway. To test for genetic epistasis between *wnt11f2*, *dact1,* and *dact2* genes we generated *wnt11f2−/−; dact1−/−; dact2*−/− triple homozygous mutants. If *wnt11f2* and *dact1/2* had independent developmental requirements, the *wnt11f2−/−; dact1−/−; dact2*−/− mutant may exhibit a phenotype distinct from *wnt11f2*−/− or *dact1−/−; dact2*−/− mutants. We found that the *wnt11f2−/−; dact1−/−; dact2*−/− triple homozygous mutant phenotype of the linear rod-like ANC was the same as the *wnt11f2*−/− mutant or *dact1−/−;dact2*−/− double mutant, without exhibiting additional or neo-phenotypes in the craniofacial cartilages or body axis (Fig. 3C,D). This result supports *dact1 and dact2* acting downstream of *wnt11f2* signaling during ANC morphogenesis, where loss of *dact1/2* function recapitulates a loss of *wnt11f2* signaling. Alternatively, both *wnt11f2* and *dact1/2* are key regulators of convergent extension during craniofacial morphogenesis, so that disruption of either the *wnt11f2* and *dact1/2* signaling results in this common morphologic endpoint.

### Lineage tracing of NCC movements in *dact1/2* mutants reveals ANC composition

The stereotypic convergent migration of cranial neural crest cells and their derivatives presents an excellent model to examine CE movements and their effects on tissue morphogenesis. The zebrafish ANC is formed from the joining of a midline frontonasal prominence derived from the anteromost cranial neural crest (NCC) cell population that migrate over the eyes and turn caudally, to join paired lateral maxillary prominences derived from the second stream of cranial NCC population that migrate rostrally (Kimmel, Miller et al. 2001, Wada, Javidan et al. 2005, Schilling and Le Pabic 2009, Dougherty, Kamel et al. 2012, Mork and Crump 2015). The ANC that forms is a planar fan-shaped structure where we and others have shown that the morphology is governed by Wnt signaling (Kimmel, Miller et al. 2001, Rochard, Monica et al. 2016).

**Figure 4.**
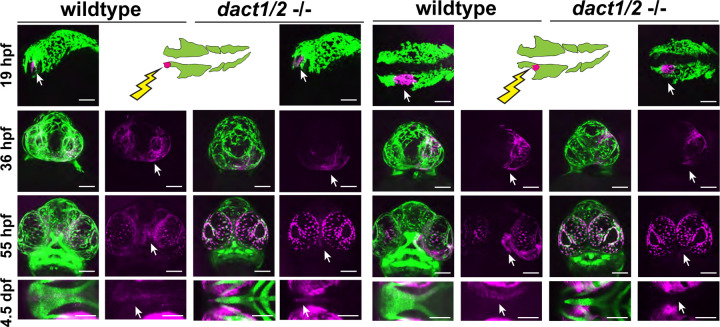
Anterior neural crest cells of the *dact1/t2*−/− mutant migrate to the midline and populate the ANC. Lineage tracing of wildtype and *dact1/2*−/− double mutant zebrafish embryos using Tg(*sox10*:kaede) line. *sox10*:kaede fluorescence is shown in green and photo-converted kaede is shown in magenta and highlighted with an arrow. Asterisks indicate that the cell population is absent. 19 hpf embryo sagittal views showing photoconversion of anterior-most neural crest population. At 36 hpf frontal images show the migration of photoconverted neural crest cells to the frontonasal prominences in wildtype and *dact1/2*−/− double mutants. At 55 hpf, frontal images show photoconverted neural crest cells populating the region of the developing ANC in wildtype and *dact1/2*−/− mutants. At 4.5 dpf ventral images show photoconverted neural crest cells populating the medial ANC in wildtype. Similarly, neural crest cells in *dact1/dact2*−/− mutants populate the rod-shaped ANC. Scale bar:100 μm.

Given the rod-like ANC we observed in the *dact1−/−; dact2*−/− double mutants, we hypothesized that the dysmorphology could be due to aberrant migration of the anteromost midline frontonasal stream of cranial NCCs or abrogated contribution from the second paired stream of maxillary NCCs. To distinguish between these possibilities, we carried out lineage tracing of the cranial NCC populations in wildtype and *dact1/2* mutants. The *dact1/2* compound mutants were bred onto a *sox10:kaede* transgenic background, where we and others have shown that the *sox10* reporter is a reliable driver of cranial neural crest labeling (Wada, Javidan et al. 2005, Dutton, Antonellis et al. 2008, Swartz, Sheehan-Rooney et al. 2011, Dougherty, Kamel et al. 2012, Kague, Gallagher et al. 2012, Mork and Crump 2015). Cranial NCC populations in wildtype and *dact1/2* mutants were targeted at 19 hpf to photoconvert Kaede reporter protein in either the anterior cranial NCCs that contribute to the frontonasal prominence, or the second stream of NCCs that contribute to the maxillary prominence, where the labeled cells were followed longitudinally over 4.5 days of development (Fig. 4). We found that the anterior NCCs of wildtype embryos migrated antero-dorsally to the eye and populated the medial ANC. To our surprise, the anterior cranial NCC also migrated to contribute to the median element of the rod-like ANC, suggesting the complex anterior then caudal migration of the anterior NCC is not disrupted by *dact1/2* mutation. This finding is in contrast to lineage tracing in another midline mutant with a similarly shaped rod-like ANC, the *syu* (*shh* null) mutant, where the anterior NCCs failed to populate the ANC (Wada, Javidan et al. 2005).

Next, the second stream of NCC population that contribute to the maxillary prominence was labeled, where they migrate and contribute to the lateral element of the ANC as expected in the wildtype (Fig 4). When the second stream of cranial CNN were labeled and followed in the *dact1/2* mutants, the cells were found to migrate normally up to 36 hpf, but did not ultimately populate the ANC in the mutant, suggesting that the morphological movements after NCC migration were disrupted by loss of *dact1/2* function. These results suggest that NCC migration itself is not regulated by *dact1/2* but that loss of *dact1/2* hinders the second stream of NCCs’ ability to populate the ANC. These results show that the rod-like ANC can be formed from 2 different NCC origins, where in the *dact1/2* mutants the ANC is contributed by the anteromost frontonasal NCCs, in contrast to the similar rod-shaped ANC of the *syu* mutants that is formed from the more posterior stream of maxillary NCCs (Wada, Javidan et al. 2005).

### Genetic interaction of *dact1/2* with Wnt regulators *gpc4* and *wls* to generate novel facial forms

Given the role of Dact/dapper as modifiers of Wnt signaling, we hypothesized that genetic interaction of *dact1/2* with *wls* and *gpc4* will modify facial morphology. Gpc4 is a glycoprotein that binds Wnt ligands and modulates Wnt signaling. *gpc4* zebrafish mutants (*kny*) have impaired convergent extension which leads to a shortened body axis (Topczewski, Sepich et al. 2001). Wls is a posttranslational modifier of Wnt ligands which promotes their secretion (Banziger, Soldini et al. 2006, Bartscherer, Pelte et al. 2006). We previously described that these components of the Wnt/PCP pathway (*gpc4* receptor, *wls* intracellular ligand chaperon, and Wnt ligands *wnt9a* and *wnt5b)* are required for craniofacial morphogenesis, where each gene affects particular morphologic aspects of chondrocytes arrangement in the cardinal axis of the ANC and Meckel’s cartilage (Rochard, Monica et al. 2016, Ling, Rochard et al. 2017). Using the ANC as a morphologic readout, we examined the genetic interaction of *dact1* and *dact2* with *wls* and *gpc4*. Compound mutants of *dact1*, *dact2, gpc4* or *wls* were generated by breeding the single alleles. Compared to wildtype ANC morphology, abrogation of *gpc4* led to increased width in the transverse axis, but shorter in the antero-posterior axis (Rochard, Monica et al. 2016). Disruption of *wls* leads to ANC morphology that is also wider in the transverse dimension, but to a lesser degree than observed in *gpc4*. Additionally, in the *wls* mutant, chondrocytes stack in greater layers in the sagittal axis (Rochard, Monica et al. 2016).

**Figure 5.**
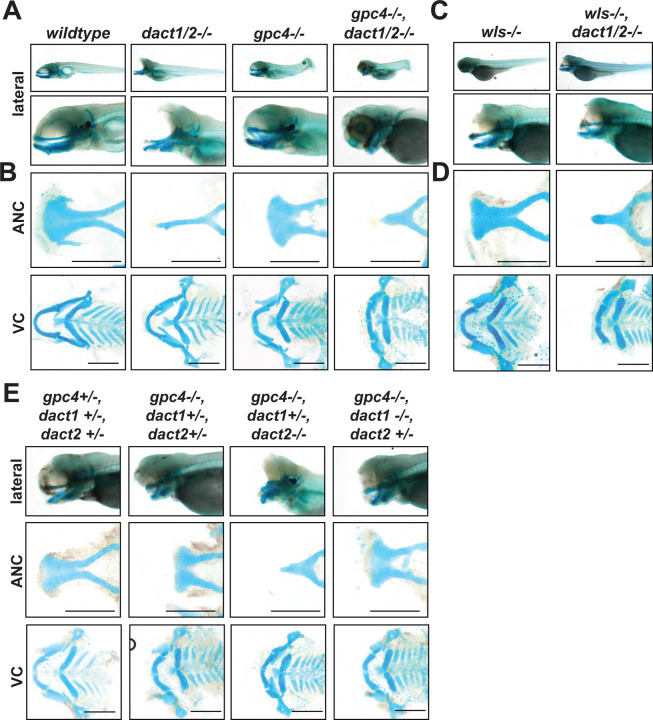
A nonoverlapping functional role for *dact1*, *dact2,* and *gpc4* and *wls*. **A)** Representative Alcian blue stained whole-mount images of wildtype, *dact1/2*−/− double mutant, *gpc4*−/− mutant, and *gpc4−/−,dact1/2*−/− triple mutants at 4 dpf. Low magnification lateral images of embryos showing tail truncation in *dact1/2*−/− mutants, shortened and kinked tail in *gpc4*−/− mutants, and a combinatorial effect in *gpc4−/−,dact1/2*−/− triple mutants. Higher magnification lateral images show a shortened midface and displaced lower jaw in *dact1/2*−/− mutants, a shortened midface in *gpc4*−/− mutant, and a combinatorial effect in *gpc4−/−,dact1/2*−/− triple mutants. **B)** Representative flat-mount images of dissected Alcian blue-stained cartilage elements. *dact1/2*−/− mutants have a narrow rod-shaped ANC while *gpc4*−/− mutants have a broad and shortened ANC. *dact1/2/gpc4* triple mutants have a combinatorial effect with a short, broad rod-shaped ANC. In ventral cartilages, *dact1/2*−/− mutants have a relatively normal morphology while Meckel’s cartilage in *gpc4*−/− mutants and *gpc4−/−,dact1/2*−/− triple mutants is truncated. **C,D)** Same as above except *wls*−/− mutant and *wls−/−,dact1/2*−/− triple mutant, with similar findings. **E)** Combinatorial genotypes of *dact1*, *dact2,* and *gpc4*. *dact2*−/− contributed the *dact/gpc4* compound phenotype while *dact1*−/− did not. Scale: 200 μm.

Disruption of *gpc4* or *wls* in addition to *dact1/2* generated ANC morphology that contained phenotypic attributes from each single mutant, so that the resultant ANC morphology represented a novel ANC form. The ANC of a triple homozygous *dact1−/−; dact2−/−; gpc4*−/− mutant was triangular, wider in the transverse axis and shorter in the antero-posterior axis compared to the rod-like ANC observed in the *dact1−/−;dact2*−/− double mutant (Fig. 5A). Similarly, the ANC of a triple homozygous *dact1−/−; dact2−/−; wls*−/− mutant was in the shape of a rod, shorter in the antero-posterior axis and thicker in the sagittal axis compared to the *dact1−/−;dact2*−/− double mutant, reflecting attributes of the *wls* mutant (Fig. 5B). In addition to the ANC phenotypes, the triple homozygous *dact1−/−; dact2−/−; gpc4*−/− mutant also had a short body axis and truncated tail similar but more severe than the *gpc4* mutant (Fig. 5A). Since compound disruption of *dact1*, *dact2*, and *gpc4* or *wls* resulted in a new phenotype we conclude that these genes function in different aspects of Wnt signaling during craniofacial development.

As we analyzed the subsequent genotypes of our *dact1/dact2/gpc4* triple heterozygote in-cross we gleaned more functional information about *dact1* and *dact2.* We found that *dact1* haploinsufficiency in the context of *dact2−/−; gpc4*−/− was sufficient to replicate the triple *dact1/dact2/gpc4* homozygous phenotype (Fig. 5C). In contrast, *dact2* haploinsufficiency in the context of *dact1−/−; gpc4*−/− double mutant produced ANC in the opposite phenotypic spectrum of ANC morphology, appearing similar to the *gpc4*−/− mutant phenotype (Fig. 5C). These results show that *dact1* and *dact2* do not have redundant function during craniofacial morphogenesis, and that *dact2* function is more indispensable than *dact1*. These results also suggest that *dact1* and *gpc4* may have overlapping roles in craniofacial development.

### *dact1/2* and *gpc4* regulate axis extension via overlapping and distinct cellular pathways

**Figure 6.**
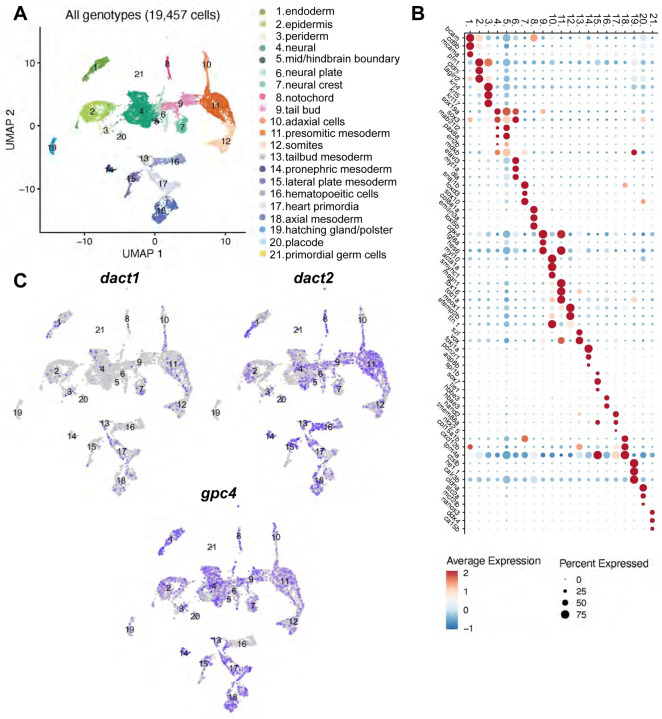
Single-cell RNAseq of 4 ss wildtype, *dact1/2*−/− mutant and *gpc4*−/− mutants. **A)** UMAP showing cluster identification. **B)** Dot plot showing the most differentially expressed genes between clusters. **C)** UMAP showing *dact1*, *dact2,* and *gpc4* expression in wildtype embryos.

*dact1* or *dact2* are required for CE and anterior-posterior axis lengthening during gastrulation (Fig. 3). We aimed to investigate the relationship of this defect to the midfacial dysmorphism of the *dact1*/*2* mutant. Phenotypic analyses of the interaction between *dact1/2* and *gpc4* (Fig. 5) indicate that *dact1/2* function modifies the Wnt pathway, but that the development processes leading to the narrowing of the ANC in the *dact1/2* double mutants cannot be explained by the same axis convergent extension defect observed the *gpc4* mutant (Topczewski, Sepich et al. 2001). As *dact1/2* mutants and *gpc4* mutants have similar CE defects during late gastrulation but disparate ANC morphologies later during craniofacial morphogenesis, we performed single-cell transcriptional analysis to compare *dact1/2* mutants, *gpc4* mutants, and wildtype embryos during the segmentation stage. Single-cell encapsulation and barcoded cDNA libraries were prepared from dissociated 3 ss wildtype, *dact1−/−;dact2*−/− compound mutant and *gpc4*−/− mutant embryos using the 10X Genomics Chromium platform and Illumina next-generation sequencing. Twenty clusters were identified using Louvain clustering and identity was assigned by reviewing cluster-specific markers in light of published expression data (Farrell, Wang et al. 2018, Farnsworth, Saunders et al. 2020, Bradford, Van Slyke et al. 2022) (Fig. 6A,B). Qualitatively, we did not observe any significant difference in cluster abundance between genotype groups. We found that *dact1*, *dact2*, and *gpc4* were detected at various levels across clusters, though *dact1* expression was lower than *dact2* (Fig. 6C), consistent with what we observed in RNA whole-mount ISH analysis (Fig. 1).

**Figure 7.**
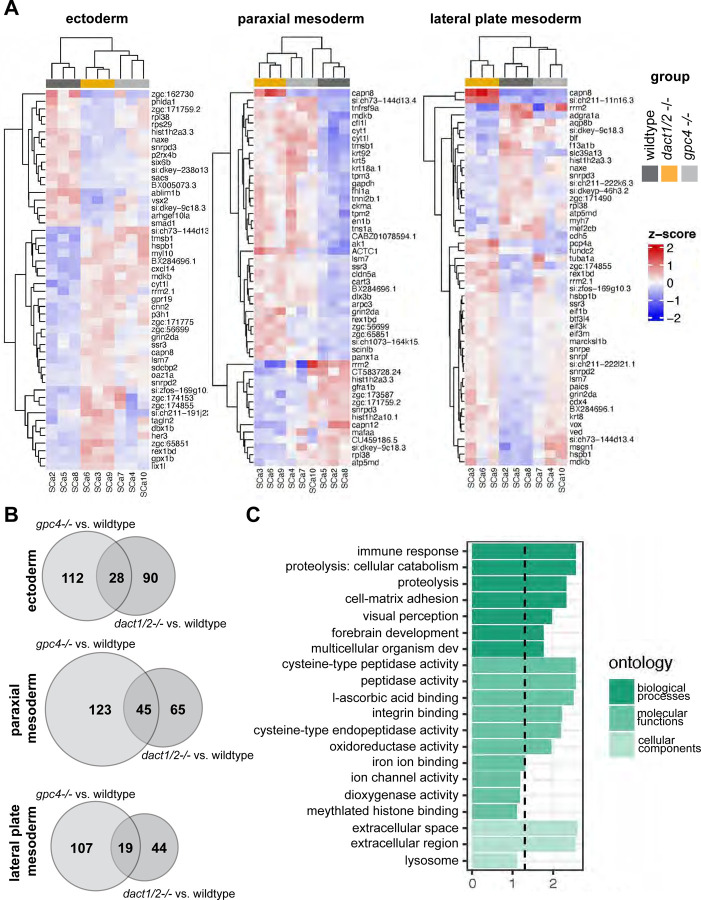
Pseudobulk differential expression analysis of single-cell RNAseq data. **A)** Heatmaps showing the 50 most differentially expressed genes in 3 major cell types; ectoderm (clusters 4,5,6,7), paraxial mesoderm (clusters 10,11,12), and lateral plate mesoderm (clusters 15, 16, 17,18) between *dact1/2*−/− mutants and wildtype and *gpc4*−/− mutants and wildtype. **B)** Venn diagrams showing unique and overlapping DEGs in *dact1/2*−/− and *gpc4*−/− mutants. **C)** GO analysis of *dact1/2*−/− mutant-specific DEGs in lateral plate mesoderm showing enrichment for proteolytic processes.

To assess the relative differences in gene expression between genotype groups, we merged clusters into broader cell lineages: ectoderm, axial mesoderm, and paraxial mesoderm (Fig. 7A). We focused on these cell types because they contribute significantly to convergent extension processes and axis establishment. For each of these cell lineages, we performed independent pseudobulk differential expression analyses (DEA) of wildtype vs. *dact1−/−;dact2*−/− mutant and wildtype vs. *gpc4*−/− mutant (Fig. 7B). In all 3 cases, we found differentially expressed genes (DEGs) that were commonly in *dact1−/−;dact2*−/− and *gpc4*−/− mutant relative to wildtype. To address the hypothesis that *dact1* and *dact2* regulate molecular pathways distinct from those regulated by *gpc4* we also identified genes that were differentially expressed only in *dact1−/−;dact2*−/− mutants or only in *gpc4*−/− mutants (Fig. 7B). Functional analysis of these DEGs found unique enrichment of intermediate filament genes in *gpc4*−/− whereas *dact1/2*−/− mutants had enrichment for pathways associated with proteolysis (Fig. S2). Enrichment for pathways associated with calcium-binding were found in both *gpc4*−/− and *dact1/2*−/−, although the specific DEGs were distinct (Fig. S2). We performed functional analyses specifically for genes that were differentially expressed in *dact1−/−;dact2*−/− mutants, but not in *gpc4*−/− mutants, and found enrichment in pathways associated with proteolysis (Fig. 7B) suggesting a novel role for Dact in embryogenesis.

**Figure 8.**
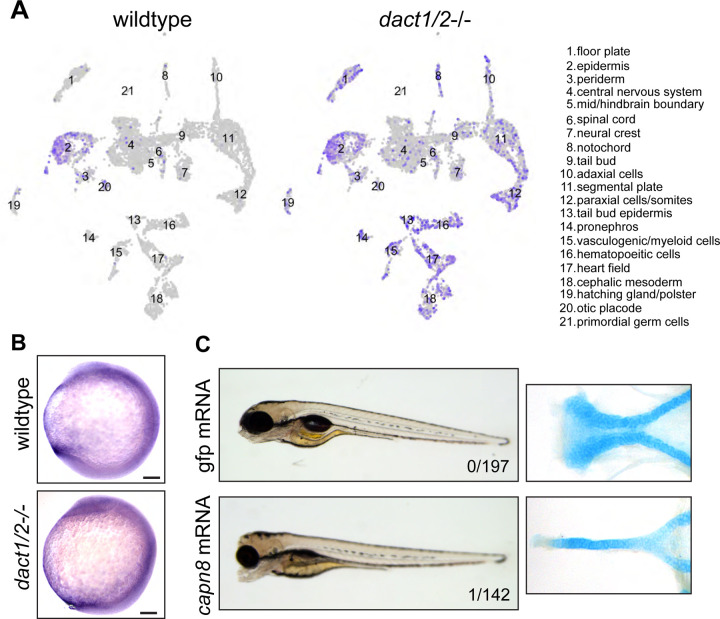
Expression of *capn8* is significantly dysregulated in *dact1/2*−/− mutants. **A)** Single-cell RNAseq gene expression analysis of *capn8* in wildtype and *dact1/2*−/− mutants. In wildtype embryos, *capn8* expression is restricted predominantly to the epidermis whereas *capn8* is widely expressed throughout the embryo in *dact1/2*−/− mutants, especially in the mesoderm. **B)** Whole-mount *in situ* hybridization of *capn8* expression in wildtype and *dact1/2*−/− mutant embryos at 2 ss. Staining corroborates the single-cell RNAseq data, with expanded ectopic expression of *capn8* throughout the embryo**. C)** Brightfield images and alcian blue staining of the ANC show ectopic expression of *capn8* mRNA (200 pg) at the 1-cell stage in wildtype embryos recapitulates the *dact1/2*−/− compound mutant craniofacial phenotype at a low frequency (1/142 injected embryos). The mutant craniofacial phenotype did not manifest in gfp mRNA (200 pg) injected 1-cell stage embryos (0/192 injected embryos).

Interrogation of *dact1−/−;dact2*−/− mutant-specific DEGs found that the calcium-dependent cysteine protease *calpain 8* (*capn8*) was significantly overexpressed in *dact1−/−;dact2*−/− mutants in paraxial mesoderm (103 fold), axial mesoderm (33 fold), and in ectoderm (3 fold; Fig. 7A). We also found that loss of *dact1/2* causes significant changes to *capn8* expression pattern (Fig. 8A). Whereas *capn8* gene expression is principally restricted to the epidermis of wildtype embryos, loss of *dact1/2* leads to significant expansion of ectopic *capn8* gene expression in broader cell types such as in mesodermal tissues (Fig. 8A). We corroborated this finding with whole mount ISH for *capn8* expression in wildtype versus *dact1/2*−/− 12 hpf embryos (Fig. 8B).

Capn8 is considered a “classical” calpain, with domain homology similar to Capn1 and 2 (Macqueen and Wilcox 2014). In adult human and mouse tissue, *Capn8* expression is largely restricted to the gastrointestinal tract (Sorimachi, Ishiura et al. 1993, Macqueen and Wilcox 2014), however embryonic expression in mammals has not been characterized. Proteolytic targets of Capn8 have not been identified, however, other classical calpains have been implicated in Wnt and cell-cell/ECM signaling (Konze, van Diepen et al. 2014), including in Wnt/Ca^+2^ regulation of convergent extension in Xenopus (Zanardelli, Christodoulou et al. 2013). To determine whether the *dact1−/−;dact2*−/− mutant craniofacial phenotype could be attributed to *capn8* overexpression, we performed injection of *capn8* mRNA into 1 cell-stage zebrafish embryos. In wildtype zebrafish, exogenous *capn8* mRNA caused the distinct *dact1/2*−/− craniofacial phenotype including a rod-like ANC at a very low frequency (1 in 142 injected embryos). This phenotype was never observed in wildtype larvae, or when wildtype embryos were injected with an equal concentration of gfp mRNA (0 in 192 injected embryos) (Fig. 8C). These findings suggest that *dact*-dependent suppression of *capn8* expression is required for normal embryogenesis and craniofacial morphogenesis.

## Discussion

In this study, we examined how *dact1* and *dact2* interact with Wnt signaling during early embryogenesis and craniofacial morphogenesis. Wnt signaling is central to the orchestration of embryogenesis and numerous proteins have been identified as modulators of Wnt signaling, including *Dact1* and *Dact2*. Several studies across *Xenopus*, zebrafish and mouse have ascribed roles to *dact1* and *dact2,* including both promoting and antagonizing Wnt signaling, depending on the developmental context. Here, we show that *dact1* and *dact2* are required for axis extension during gastrulation and show a new example of CE defects during gastrulation associated with craniofacial defects. Our data supports the hypothesis that CE gastrulation defects are not causal to the craniofacial defect of medially displaced eyes and midfacial hypoplasia and that an additional morphological process is disrupted. We show that disruption of both *dact1* and *dact2* are required to generate the dysmorphic craniofacial phenotype, as single mutants develop normally. However, based on gene expression and epistasis experiments, it is clear that these paralogs are not redundant and have unique functions. We observed that *dact1* and *dact2* have distinct spatiotemporal expression patterns throughout embryogenesis, suggesting unique roles for each paralog in developmental processes. We found that *dact1* and *dact2* contribute to axis extension, and their compound mutants exhibit a CE defect. This finding aligns with previous studies that have implicated *dact1* and *dact2* in non-canonical wnt signaling and regulation of embryonic axis extension. Based on the data, we posit that *dact1* expression in the mesoderm is required for dorsal CE during gastrulation through its role in noncanonical Wnt/PCP signaling, similar to the defect observed upon *gpc4* disruption. Conversely, we propose *dact2* functions in the prechordal mesoderm, anterior neural plate and pollster to promote anterior migration during gastrulation, a function which has also been ascribed to *wntllf2*. It is only upon loss of both functions of *dact1* and *dact2* that the craniofacial defect manifests.

Our results underscore the crucial roles of *dact1* and *dact2* in embryonic development, specifically in the connection between CE during gastrulation and ultimate craniofacial development. Our analysis of CNCC migration and contribution to the anterior neurocranium in *dact1−/−;dact2*−/− compound mutant suggests that embryonic fields determined during gastrulation effect the CNCC ability to contribute to the craniofacial skeleton. It will be important to test the generality of this phenomenon utilizing other gastrulation mutants and other model systems.

By comparing the transcriptome of a CE mutant, i.e. *gpc4*−/− with that of the *dact1−/−;dact2*−/− compound mutant, we identified a novel role for *dact1/2* in the regulation of proteolysis, with significant misexpression of *capn8* in the mesoderm of *dact1−/−;dact2*−/− mutants. Although at a very low frequency, ectopic expression of *capn8* mRNA recapitulated the *dact1−/−;dact2*−/− mutant craniofacial phenotype, suggesting that inhibition of *capn8* expression in the mesoderm by dact is required for normal morphogenesis. Genes involved in calcium ion binding (i.e. Purkinje cell protein 4 (*pcp4a*)) were also differentially expressed in the *dact1−/−;dact2*−/− mutants and we predict that altering intracellular calcium handling in conjunction with *capn8* overexpression would increase the frequency of the recapitulated *dact1−/−;dact2*−/− mutant phenotype.

Capn8 is described as a stomach-specific calpain and a role during embryogenesis has not been previously described. Calpains are typically calcium-activated proteases and it is feasible that Capn8 is active in response to Wnt/Ca^2+^ signaling. A close family member, Capn2 has been found to modulate Wnt signaling by degradation of beta-catenin (Zanardelli, Christodoulou et al. 2013, Konze, van Diepen et al. 2014). These findings suggest that dact-dependent suppression of *capn8* expression is necessary for normal embryogenesis and craniofacial morphogenesis, further expanding the functional repertoire of *dact1/2*. Further research is required to examine the direct regulatory role of dacts on *capn8* expression. Our findings also warrant investigation into the role of *capn8* during embryogenesis and possible implications for known craniofacial or other disorders.

Another gene differentially expressed specifically in the *dact1−/−,dact2*−/− embryos is *smad1*. Smad1 acts in the TGF-β signaling pathway and dact2 has been described to inhibit TGF-β and Nodal signaling by promoting the degradation of Nodal receptors (Zhang, Zhou et al. 2004, Su, Zhang et al. 2007, Meng, Cheng et al. 2008, Kivimae, Yang et al. 2011). It is robustly feasible that dysregulation of TGF- β signaling in the *dact1*−/−;*dact2*−/− mutant contributes to the craniofacial phenotype. Future research will examine the role of *dact1* and *dact2* in the coordination of Wnt and TGF-β signaling and the importance of this coordination in the context of craniofacial development.

The zebrafish has proven to be an important tool in studying early developmental biology. Early days of forward genetic screens, careful descriptive analyses, and gene epistasis experiments were seminal to our understanding of the key regulators of development and embryogenesis, including the Wnt pathway. With the substantial technological advances that have become available, including gene regulation analyses, transcriptomics, and advanced real-time imaging, we can revisit those initial discoveries and expand the breadth and depth of scientific knowledge. Moving forward we are positioned to make progress in our understanding of the complexities of spatial and temporal regulation of the key developmental signaling pathways as well as discover how these different pathways interact with each other.

## Methods

### Animals and gene editing

All animal husbandry and experiments were performed in accordance with approval from Massachusetts General Hospital Institutional Animal Care and Usage Committee. Zebrafish (*Danio rerio*) embryos and adults were maintained in accordance with institutional protocols. Embryos were raised at 28.5 C in E3 medium (Carroll, Macias Trevino et al. 2020) and staged visually and according to standardized developmental timepoints (Westerfield 1993). All zebrafish lines used for experiments and gene editing were generated from the Tubingen or AB strain. The *wnt11f2* mutant line and gpc4−/− mutant line were obtained from ZIRC (wnt11f2^tx226/+^ and gpc4^hi1688Tg/+^ respectively). The *wls*−/− mutant line was originally gifted to the lab and independently generated, as previously described (Rochard, Monica et al. 2016). The *sox10*:kaede transgenic line was previously generated and described by our lab (Dougherty, Kamel et al. 2012).

CRISPR sgRNA guides were designed using computational programs ZiFiT Targeter v4.2 (zifit.partners.org/ZiFit) (Sander, Zaback et al. 2007), crispr.mit.edu (https://zlab.bio/guide-design-resouces) (Ran, Hsu et al. 2013), and ChopChop (https://chopchop.cbu.uib.no (Montague, Cruz et al. 2014) with traditional sequence constraints. Guides were chosen that were predicted to give high efficiency and specificity and located near the 5’ end of the gene. Guides best meeting these parameters were selected in exon 2 of *dact1* and exon 4 of *dact2*. No suitable gRNA with sufficient efficiency were identified for *dact2* 5’ of exon 4 and the resulting phenotype was reassuring compared to previous morpholino published results. Guides for *dact1* and *dact2* and Cas9 protein were prepared and microinjected into 1 cell-stage zebrafish embryos and founders were identified as previously described (Carroll, Macias Trevino et al. 2020). Primers flanking the sgRNA guide site were designed for genotyping and fragment analysis was performed on genomic DNA to detect base pair insertion/deletion. Sanger sequencing was performed to verify targeted gene mutation and confirm the inclusion of a premature stop codon.

### Microinjection of mRNA

mRNA for injection was generated using an *in vitro kit* (Invitrogen mMessage mMachine) and cloned cDNA as a template. One-cell stage zebrafish embryos were injected with 2nL mRNA solution.

### Whole-mount and RNAscope *in situ* hybridization

Whole-mount *in situ* hybridization (WISH) was performed as previously described (Carroll, Macias Trevino et al. 2020). Zebrafish embryonic cDNA was used as a template to generate riboprobes. Primers were designed to PCR amplify the specific riboprobe sequence with a T7 promoter sequence linked to the reverse primer. *In vitro* transcription was performed using a T7 polymerase and DIG-labeled nucleotides (Roche). RNAscope was performed on sectioned zebrafish larvae as previously described (Carroll, Macias Trevino et al. 2020). Probes were designed by and purchased from ACD Bio. Hybridization and detection was performed according to the manufacturer’s protocol. Sections were imaged using a confocal microscope (Leica SP8) and z-stack maximum projections were generated using Fiji software.

### Alcian blue staining and imaging

Alcian blue staining and imaging were performed at 4 dpf as previously described (Carroll, Macias Trevino et al. 2020). Briefly, larvae were fixed in 4% formaldehyde overnight at 4C. Larvae were dehydrated in 50% ethanol and stained with Alcian blue as described (Walker and Kimmel 2007). Whole and dissected larvae were imaged in 3% methylcellulose using a Nikon Eclipse 80i compound microscope with a Nikon DS Ri1 camera. Z-stacked images were taken and extended depth of field was calculated using NIS Element BR 3.2 software. Images were processed using Fiji software.

### Axis measurements

Compound *dact1*+/−; *dact2*+/− zebrafish were in-crossed and progeny were collected from 2 separate clutches and fixed in 4% formaldehyde at approximately 8ss. Embryos were individually imaged using a Zeiss Axiozoom stereoscope and processed for DNA extraction and genotyping (Westerfield 1993). Images were analyzed using Fiji. A circle was drawn to overlay the yolk and the geometric center was determined using the function on Fiji. Using the Fiji angle tool, lines were drawn from the center point to the anterior-most point of the embryo and from the center to the posterior-most point of the embryo. The resulting inner angle of these lines was determined. Each angle measurement was then calculated as a ratio to the average angle of the wildtype embryos. ANOVA was performed to determine statistical significance, p<0.05.

### Single-cell RNA sequencing (scRNA-seq)

Single-cell transcriptomic analyses were performed on 10 zebrafish embryos, including 4 wildtype, 3 *dact1−/−;dact2*−/− compound mutant, and 3 *gpc4*−/− mutant embryos. Embryos were collected at 3 ss with *dact1−/−,dact2*−/− and *gpc4*−/− mutants being identified by their truncated body axis. Embryos were dechorionated with a short (approximately 10 min) incubation in 1 mg/ml Pronase and then washed 3x in embryo medium. Cell dissociation was performed with modifications as previously described (Farrell, Wang et al. 2018). Each embryo was transferred to 50 μl DMEM/F12 media on ice. To dissociate cells, media was replaced with 200 μl DPBS (without Ca^2+^ and Mg^2+^) with 0.1% BSA. Embryos were disrupted by pipetting 10x with a P200 pipette tip. 500 μl of DPBS + 0.1% BSA was added and cells were centrifuged at 300xg for 1 min. Cell pellets were resuspended in 200 μl DPBS + 0.1% BSA and kept on ice. Just prior to encapsulation, cells were passed through a 40 μm cell strainer, and cell counts and viability were measured. After droplet encapsulation, barcoding, and library preparation using the 10X Genomics Chromium Single Cell 3’ kit (version 3), data were sequenced on an Illumina NovaSeq 6000 sequencer.

FASTQ files were demultiplexed and aligned to the GRCz11 build of the zebrafish genome using Cellranger (version 6.1.0) (Zheng, Terry et al. 2017). Raw Cellranger count matrices were imported into R (version 4.1.2) using Seurat (version 4.1.0) (Hao, Hao et al. 2021). First, we reviewed data for quality and excluded any droplet that did not meet all of the following criteria: (i) have at least 1,500 unique molecular identifiers (UMIs), (ii) covering at least 750 distinct genes; (iii) have <5% of genes mapping to the mitochondrial genome; and (iv) have a log10 of detected genes per UMI >80%. After quality control, the dataset was also filtered to exclude genes with a detection rate below 1 in 3,000 cells, leaving a total of 20,078 distinct genes expressed across 19,457 cells for analysis.

The quality-controlled count data were normalized using Pearson’s residuals from the regularized negative binomial regression model, as implemented in Seurat::SCTransfrom (Hafemeister and Satija 2019). When computing the SCT model, the effect of the total number of UMIs and number of detected genes per cell were regressed out. After normalization, the top 3,000 most variably expressed genes were used to calculate principal components (PCs). Data were then integrated by source sample using Harmony (version 0.1.0) (Korsunsky, Millard et al. 2019). A two-dimensional uniform manifold approximation and projection (UMAP) (Becht, McInnes et al. 2018) was then derived from the first 40 Harmony embeddings for visualization. Using the integrated Harmony embeddings, clusters were defined with the Louvain clustering method, as implemented within Seurat. A resolution of 0.3 was used for cluster definition. Cluster identities were assigned by manually reviewing the results of Seurat::FindAllMarkers, searching for genes associated to known developmental lineages. Gene expression data for key markers that guided cluster identity assignment were visualized using Seurat::DotPlot.

Following this detailed annotation, some clusters were grouped to focus downstream analyses on 3 major lineages: ventral mesoderm (grouping cells from the pronephros, vasculogenic/myeloid precursors, hematopoietic cells, heart primordium, and cephalic mesoderm clusters), dorsal mesoderm (adaxial cells, segmental plate, and paraxial mesoderm), ectoderm (CNS, mid/hindbrain boundary, spinal cord, and neural crest). For those 3 lineages, single-cell level data were aggregated per sample and cluster to perform pseudobulk differential expression analyses (DEA) contrasting genotypes. Independent pairwise comparisons of *dact1−/−;dact2*−/− versus wildtype and *gpc4*−/− vs wildtype were performed using DESeq2 (v1.34.0) (Love, Huber et al. 2014). P-values were corrected for multiple testing using the default Benjamini-Hochberg method; log2 fold change values were corrected using the apeglm shrinkage estimator (Zhu, Ibrahim et al. 2019). Significance was defined as an adjusted p-value < 0.1 and log2 fold change > 0.58 in absolute value. Heatmaps of the top most significant differentially expressed genes were generated from the regularized log transformed data using pheatmap (version 1.0.12). Overlap in significant genes across pairwise comparisons were determined and visualized in Venn diagrams. Over-representation analyses against the Gene Ontology (GO) database were ran using clusterProfiler (version 4.2.2) (Wu, Hu et al. 2021), using as input the set of genes found to be differentially expressed in the comparison of *dact1−/−;dact*−/− versus wildtype but not *gpc4*−/− versus wildtype. Sequencing data have been deposited in GEO under accession code GSE240264.

### Statistical analysis

Analyses were performed using Prism Software (GraphPad) unless otherwise specified. An unpaired Student’s *t* test or one-way ANOVA with multiple comparisons was used as indicated and a *P*-value <0.05 was considered significant. Graphs represent the mean +/− the SEM and *n* represents biological replicates.

## Supplementary Material

Supplement 1 **Figure S1.** Characterization of CRISPR/Cas9 generated *dact1*−/− and *dact2*−/− mutants. **A)** Schematic representations of *dact1* and *dact2* exons, positions of guide RNA target site, introduced premature stop codon (arrow), and sequences of mutations. **B)** Expression levels of *dact1* and *dact2* mRNA by RT-qPCR in 12 hpf *dact1*−/− mutants, *dact2*−/− mutants, and *dact1/2*−/− compound mutants. 8 embryos were pooled for mRNA isolation per sample. **C)** Injection of *dact1* mRNA, *dact2* mRNA, or a combination of *dact1* and *dact2* mRNA rescues the rod-shaped ANC phenotype in *dact1/2*−/− compound mutants. Representative images of Alcian blue stained *dact1/2*−/− double mutant treated with 300 pg *dact1* mRNA and 300 pg *dact2* mRNA. Arrow highlights normal ANC. **D)** Quantification of the mutant craniofacial phenotype observed in a *dact1−/−,dact2*+/− breeding in-cross. Without mRNA injection, the mutant phenotype was observed at approximately the expected Mendelian ratio of 25%. Injection with *dact1* mRNA, *dact2* mRNA, or a combination of *dact1* and *dact2* mRNA decreased the frequency that the mutant craniofacial phenotype was observed.
**Figure S2.** Loss of *gpc4* and loss of *dact1/2* leads to distinct changes in gene expression profiles but with some overlapping functions. GO analysis of DEGs identified between *gpc4*−/− and wildtype embryos and *dact1/2*−/− and wildtype embryos identified changes in calcium ion binding and actin interaction in both mutants.
