# Supplementary figures and images for: dact1/2 modifies noncanonical Wnt signaling and calpain 8 expression to regulate convergent extension and craniofacial development
Source: bioRxiv. 2023 Nov 7:2023.11.07.566024. Preprint. [Version 1] doi: 10.1101/2023.11.07.566024 (PMC10659360; doi:10.1101/2023.11.07.566024)

**A**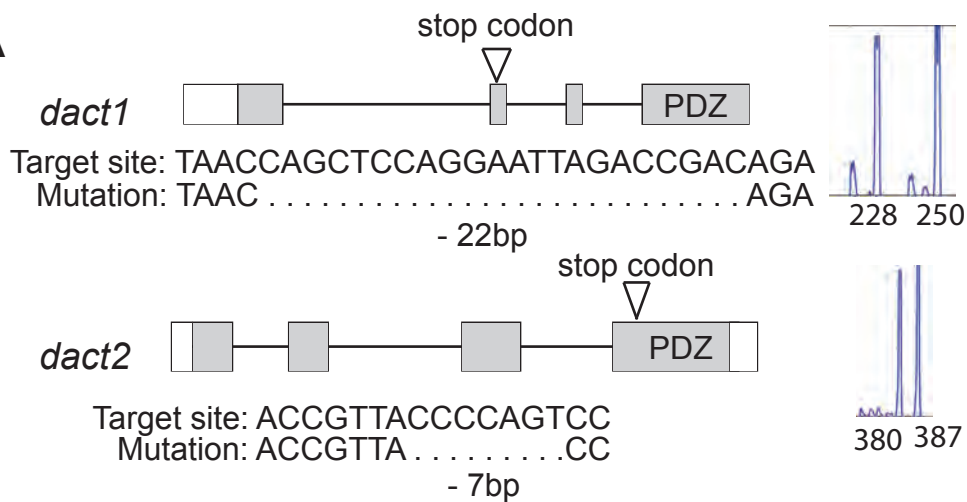**B**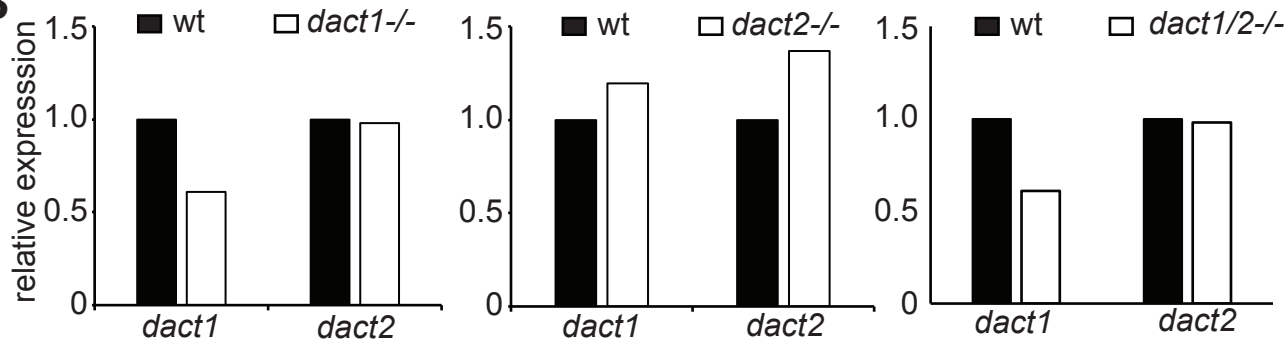**C**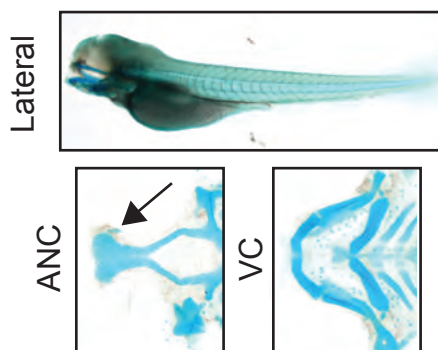**D**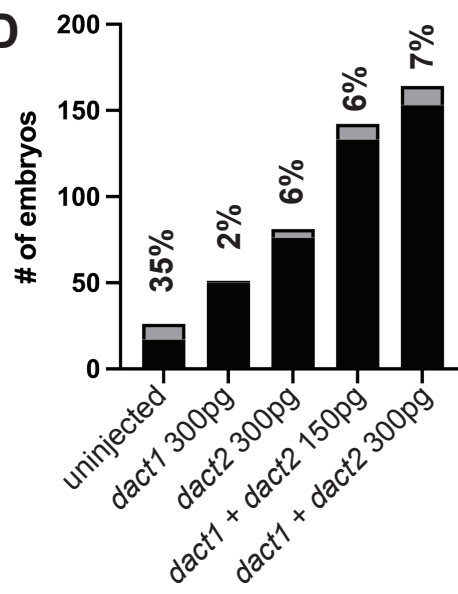

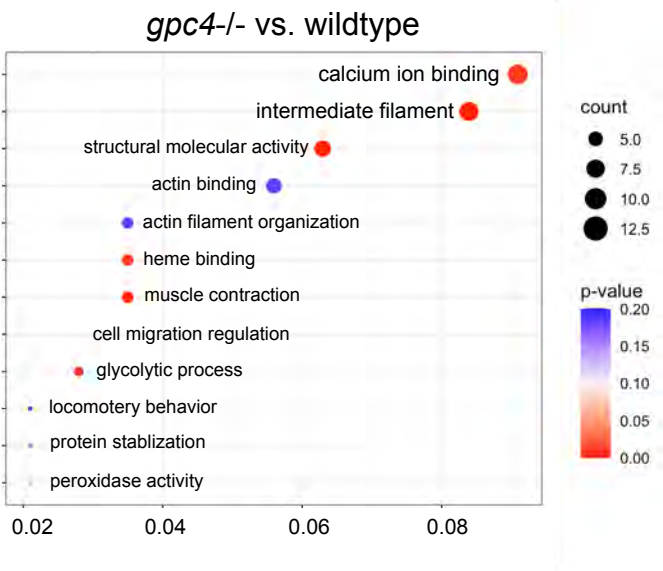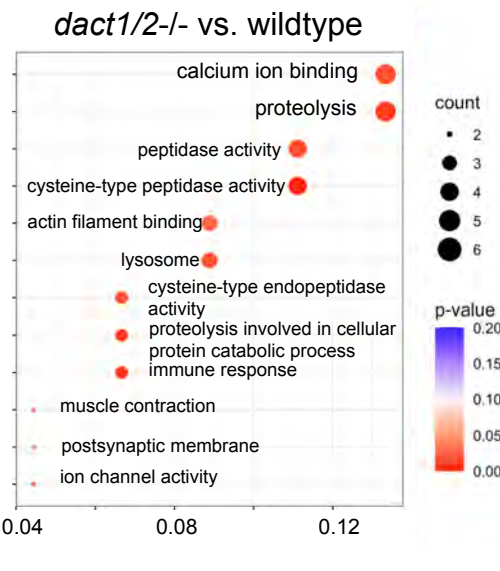

Supplement: Supplement 1 — Figure S1. Characterization of CRISPR/Cas9 generated dact1−/− and dact2−/− mutants. A) Schematic representations of dact1 and dact2 exons, positions of guide RNA target site, introduced premature stop codon (arrow), and sequences of mutations. B) Expression levels of dact1 and dact2 mRNA by RT-qPCR in 12 hpf dact1−/− mutants, dact2−/− mutants, and dact1/2−/− compound mutants. 8 embryos were pooled for mRNA isolation per sample. C) Injection of dact1 mRNA, dact2 mRNA, or a combination of dact1 and dact2 mRNA rescues the rod-shaped ANC phenotype in dact1/2−/− compound mutants. Representative images of Alcian blue stained dact1/2−/− double mutant treated with 300 pg dact1 mRNA and 300 pg dact2 mRNA. Arrow highlights normal ANC. D) Quantification of the mutant craniofacial phenotype observed in a dact1−/−,dact2+/− breeding in-cross. Without mRNA injection, the mutant phenotype was observed at approximately the expected Mendelian ratio of 25%. Injection with dact1 mRNA, dact2 mRNA, or a combination of dact1 and dact2 mRNA decreased the frequency that the mutant craniofacial phenotype was observed. Figure S2. Loss of gpc4 and loss of dact1/2 leads to distinct changes in gene expression profiles but with some overlapping functions. GO analysis of DEGs identified between gpc4−/− and wildtype embryos and dact1/2−/− and wildtype embryos identified changes in calcium ion binding and actin interaction in both mutants. [file NIHPP2023.11.07.566024v1-supplement-1.pdf]
